# Reformulating the Quantum Uncertainty Relation

**DOI:** 10.1038/srep12708

**Published:** 2015-08-03

**Authors:** Jun-Li Li, Cong-Feng Qiao

**Affiliations:** 1 Department of Physics, University of the Chinese Academy of Sciences, YuQuan Road 19A, Beijing 100049, China; 2 CAS Center for Excellence in Particle Physics, Beijing 100049, China

## Abstract

Uncertainty principle is one of the cornerstones of quantum theory. In the literature, there are two types of uncertainty relations, the operator form concerning the variances of physical observables and the entropy form related to entropic quantities. Both these forms are inequalities involving pairwise observables, and are found to be nontrivial to incorporate multiple observables. In this work we introduce a new form of uncertainty relation which may give out complete trade-off relations for variances of observables in pure and mixed quantum systems. Unlike the prevailing uncertainty relations, which are either quantum state dependent or not directly measurable, our bounds for variances of observables are quantum state independent and immune from the “triviality” problem of having zero expectation values. Furthermore, the new uncertainty relation may provide a geometric explanation for the reason why there are limitations on the simultaneous determination of different observables in *N*-dimensional Hilbert space.

The uncertainty principle is one of the most remarkable characteristics of quantum theory, which the classical theory does not abide by. The first formulation of the uncertainty principle was achieved by Heisenberg^1^, that is the renowned inequality Δ*x*Δ*p* ≥ *ħ*/2, which comes from the concept of indeterminacy of simultaneously measuring the canonically conjugate quantities position and momentum of a single particle. Later, Robertson generalized this uncertainty relation to two arbitrary observables *A* and *B* as^2^


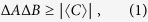


where the standard deviation, i.e. the square root of the variance, is defined to be 
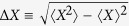
 for observables *X* and the commutator 2*iC* = [*A*, *B*]≡  *AB* − *BA*. The relation (1) reflects that the standard deviations of *A* and *B* are bounded by the expectation value of their commutator in a given quantum state of the system. However, this expectation value can be zero, even for observables that are incompatible, and makes the inequality trivial. To address this problem the uncertainty relation was expressed in terms of Shannon entropies^3^,^4^, where an improved version takes the following form^5^


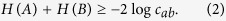


Here *H*(*A*) is the Shannon entropy of the probability distribution of the eigenbasis 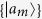 in the measuring system, and similarly is the *H*(*B*). The bound 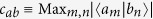 is the eigenbases’ maximum overlap of operators *A* and *B*, and therefore is independent of the state of system.

The progress in the study of uncertainty relation has profound significance on the formalism of quantum mechanics (QM) and far reaching consequences in quantum information sciences, e.g., providing the quantum separability criteria^6^, determining the quantum nonlocality^7^,^8^ (see for example Ref. 9 for a recent review). Therefore, the uncertainty principle has been the focus of modern physics for decades.

For variance-based uncertainty relations, improvements designated for mixed states had been proposed with strengthened but state dependent lower bounds^10^,^11^. Despite the progress on getting stronger uncertainty relations^12^,^13^, the problem that the lower bounds depend on the state of the system remains^14^. On the other hand, by proposing new measures of uncertainties similar as that of entropy, state independent lower bounds could be obtained^15^. There was also the combination approach involving both the entropic measures and variances in a single uncertainty relation, where only a nearly optimal lower bound could be derived^16^. Hence, obtaining the state independent optimal trade-off uncertainty relation for variances of physical observables is still an urgent and open question.

In this work, we present a new type of uncertainty relation for multiple physical observables, which is applicable in cases of both pure and mixed quantum states. Our strategy to obtain the uncertainty relation includes three steps: first decompose the quantum state of system and physical observables in Bloch space; then express the variances of observables as functions of relative angles between Bloch vectors; last, apply triangle inequalities to these angles to get constraint functions for the variances of observables, which may remarkably give out the state independent optimal trade-off uncertainty relations.

## Results

### Variances in form of Bloch vectors

In quantum theory, the systems are generally described by density matrices, which are Hermitian, and physical observables are represented by operator matrices, which are also Hermitian. The uncertainty of an observable *A* for the physical system represented by matrix *ρ* is measured by the variance


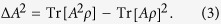


Here Tr is the trace of a matrix. Note that the variance Δ*A*^2^ are invariant under a substraction of constant diagonal matrix from *A*, i.e., Δ(*A* − *αI*)^2^ = Δ*A*^2^ with *α* being any real number and *I* is the identity matrix. Therefore, without loss of generality we are legitimate to consider observables of traceless Hermitian operators.

The *N* × *N* unitary matrices with determinant 1 form the special unitary group of degree *N*, denoted by SU(*N*). There are *N*^2^ − 1 traceless Hermitian matrices λ_*j*_ of dimension *N* × *N*, which constitute the generators of SU(*N*) group


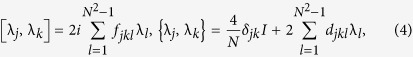


where the bracket represents the commutator and the anti-commutator is defined to be {*A*, *B*} ≡  *AB* + *BA*; *d*_*jkl*_ and *f*_*jkl*_ are symmetric and anti-symmetric structure constants of SU(*N*) group, respectively. Any *N* × *N* Hermitian matrix may be decomposed in terms of these generators^17^, including quantum states and system observables, i.e.


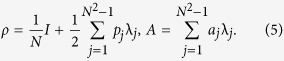


Here, *p*_*j*_ = Tr[*ρλ*_*j*_] and 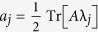. In this form, the *N* × *N* Hermitian matrices *A* and *ρ* may be represented by the *N*^2^ − 1 dimensional real vectors 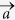 and 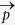 with the components of *a*_*j*_ and *p*_*j*_. This is known as the Bloch vector form of the Hermitian matrix^18^ and the norms of vectors 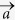 and 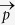 are 
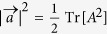
 and 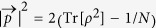, respectively. The quantity 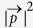 may be regarded as a measure of the degree of pureness of quantum state. For pure state 
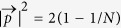
, while for completely mixed state 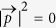.

Substituting (5) into (3), the variance of observable *A* for the quantum state *ρ* may be rewritten as


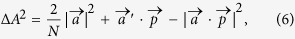


with 
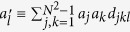
. The variance now is completely characterized by the angles between the vector 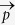 associated with the quantum state *ρ* and vectors 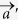 and 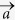 associated with the Hermitian operator *A*, i.e., 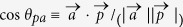, 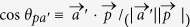. In Methods section, a general configuration for Bloch vectors of variances will be given.

In 2-dimensional Hilbert space, the Bloch vector forms of the quantum state *ρ* and observables *A*, *B*, and *C* may be represented by 3-dimensional real vectors 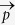, 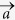, 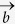, 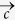, respectively. In this case, the SU(2) generators λ_*j*_ are Pauli matrices *σ*_*i*_, *i* = 1, 2, 3. From equation (6), the variances of *A*, *B*, and *C* become


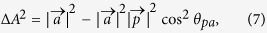



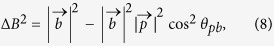



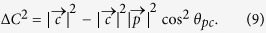


**Figure 1:**
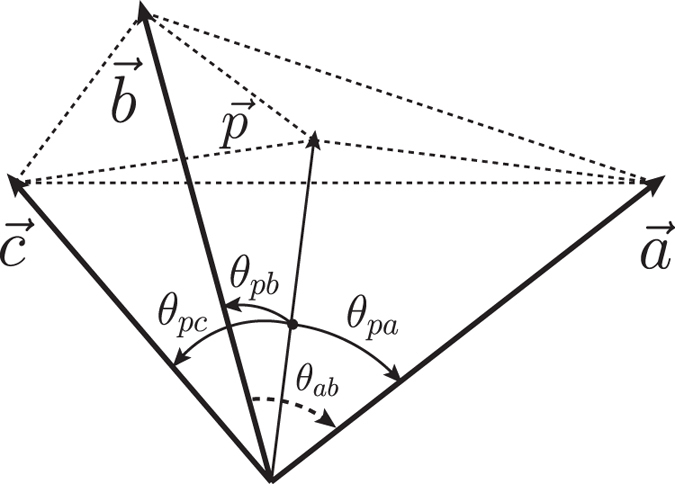
The geometric relation between the quantum state 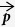 and the observables 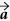, 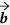, and 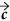 in 3-dimensional real space. The angles between 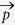 and 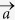 and 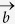 satisfy 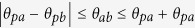. There are only two free angles in *θ*_*pa*_, *θ*_*pb*_, and *θ*_*pc*_ because 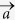, 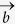, and 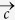 are 3-dimensional real vectors.

Here, *θ*_*pa*_, *θ*_*pb*_, and *θ*_*pc*_ are the angles between 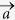, 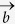, 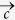, and 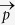, see Fig. 1. Note that 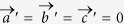 due to the fact that the symmetric structure constants are all zero in SU(2). As the inversion of a vector, e.g., 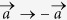, does not change the value of the observable variance, we may choose *θ*_*pa*_, *θ*_*pb*_, *θ*_*pc*_ ∈ [0, *π*/2].

### The uncertainty relations for general qubit systems

For two observables *A* and *B* and quantum state *ρ*, there exist the following triangular inequalities for *θ*_*pa*_, *θ*_*pb*_, and *θ*_*ab*_ (the angle between 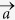 and 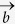, see Fig. 1):


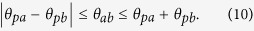


Performing cosine to equation (10) and using equations (7) and (8), we have the following theorem:

**Theorem 1** *For a qubit system, there exists the following uncertainty relation for arbitrary observables A and B*

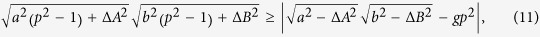
*with* 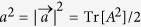, 
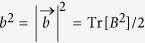
, 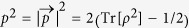, *and* 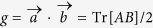.

The theorem applies to both pure and mixed states of the qubit system, and the equality may be obtained when the Bloch vector of the quantum state 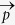 is coplane with that of the observables 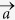 and 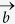.

For the completely mixed states of *ρ* = *I*/2, we have *p*^2^ = 0 and equation (11) leads to


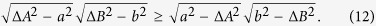


This is equivalent to that Δ*A*^2^ = *a*^2^, Δ*B*^2^ = *b*^2^. For pure states of *P*^2^ = 1, equation (11) reduces to


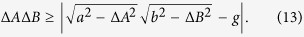


If we further assume 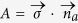 and 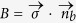, where 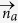 and 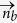 are arbitrary unit vectors, then


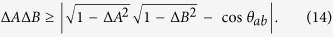


**Figure 2:**
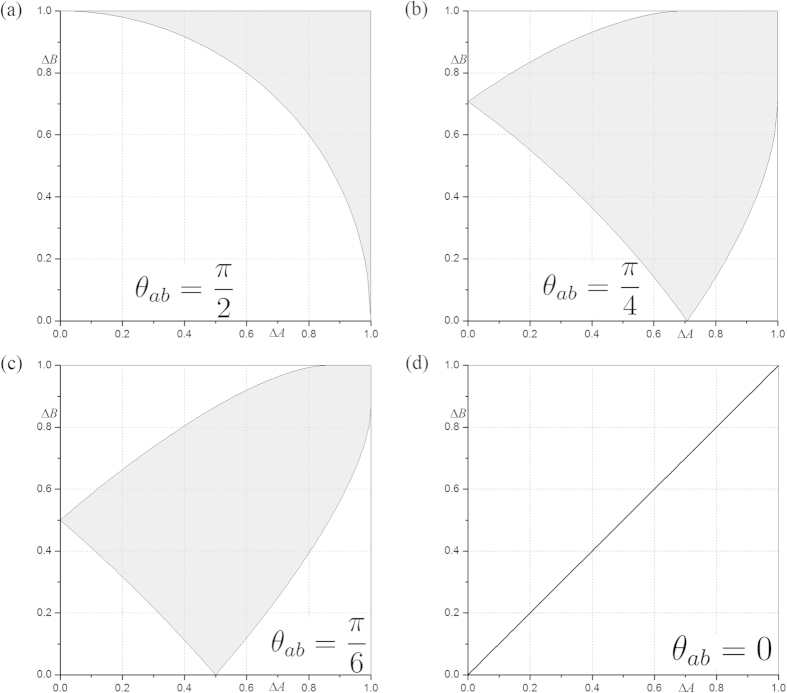
The trade-off relations between the variances of 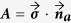 and 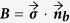 for different angles *θ*_*ab*_ between 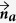 and 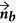. The shaded regions correspond to the allowed values of the variances when (**a**) *θ*_*ab*_ = *π*/2, (**b**) *θ*_*ab*_ = *π*/4, (**c**) *θ*_*ab*_ = *π*/6, and (**d**) *θ*_*ab*_ = 0 where a line of Δ*B* = Δ*A* is obtained.

Here *θ*_*ab*_ is the angle between 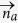 and 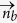. Figure 2 illustrates the trade-off relations between the variances of Δ*A*^2^ and Δ*B*^2^ for four different values of *θ*_*ab*_.

To compare with the existing uncertainty relations in the market, we exploit a recent appeared uncertainty relation with state dependent lower bound as an example^13^. It reads,


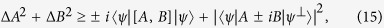


with 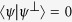. Suppose in qubit system 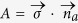, 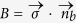, Δ*A*^2^ = 1/4 and the angles between observables *A* and *B* is *π*/6, equation (15) then tells


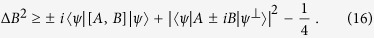


While our constraint relation (14) gives


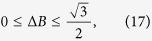


which can be read directly from Fig. 2(c). Our result (17) is quantum state independent and gives not only the lower bound, but also a span for Δ*B*, which is obviously superior to (16). Moreover, generally speaking the equation (15) is not applicable to mixed states 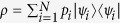 where *p*_*i*_ > 0 and 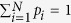, since there is no quantum state that could be orthogonal to all the 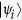 in 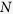-dimensional system^13^.

When three obervables *A*, *B*, and *C* are considered, their variances under the quantum state *ρ* are characterized by three angles *θ*_*pa*_, *θ*_*pb*_, and *θ*_*pc*_, see also Fig. 1. Because only two of these angles are free in 3-dimensional real space, we have the following proposition:

**Proposition 1** *For three independent observables in 2-dimensional Hilbert space, the trade-off relation for the variances of observables turn out to be an equality.*

As the validity of Proposition 2 is quite obvious, we only present a simple example as a demonstration of proof. Suppose three observables are *A* = *σ*_1_, *B* = *σ*_1_ cos *θ*_*ab*_ + *σ*_2_ sin *θ*_*ab*_, and *C* = *σ*_3_, or in the Bloch vector form 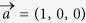, 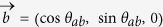 and 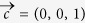. An arbitrary quantum state may be constructed as 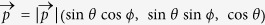, where *θ*, *ϕ* are the polar and azimuthal angles in the 3-dimensional real space. For pure states of 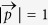, substituting the values of cos *θ*_*pa*_, cos *θ*_*pb*_, and cos *θ*_*pc*_ into equations (7, 8, 9), one then has the following trade-off relation for Δ*A*^2^, Δ*B*^2^, and Δ*C*^2^,


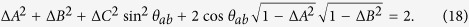


Here, *θ*_*ab*_ will always be a constant as long as the observable *B* is given. It is interesting to observe that the uncertainty relation of equation (14) can be obtained by projecting the “certainty” relation (18) onto the Δ*A*-Δ*B* plane with 0 ≤ Δ*C*^2^ ≤ 1.

In general, by expressing quantum states and physical observables in Bloch space, the state independent uncertainty relation involving several observables may be constructed. For the pure qubit system, the variances of incompatible observables (not only pairwise) cannot be zero simultaneously, due to the fact that the quantum state of system in the vector form 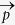 cannot simultaneously parallel to those unparallel vectors (incompatible observables, 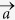, 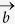, and 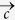). This pictorial illustration is quite instructive, which gives the succinct geometrical account for the uncertainty relations of variances.

## Discussion

There are two types of relations pertaining to the uncertainty principle, i.e., the uncertainty relation and the measurement disturbance relation (MDR)^19^. While the uncertainty relation involves the ensemble properties of variances, the MDR relates the measurement precision to its back actions, which is currently a hot topic^20^. Though being fundamentally different, the uncertainty relation and the MDR are both shown to be correlated with the quantum nonlocality^8^,^21^. Therefore, it is expected that every new forms of uncertainty relations may shed some light on the study of the connection between uncertainty principle and quantum nonlocality.

To summarize, we presented a new type of uncertainty relation which completely characterizes the trade-off relations among the variances of several physical obervables for both pure and mixed quantum systems. It provides the state independent optimal bounds not only for the variances of pairwise incompatible observables, but also for the multiple incompatible observables. Unlike the prevailing uncertainty relations in the literature, our bounds for the variances of observables are immune from the “triviality” problem of having null expectation value. As a heuristic example, we showed, geometrically, that our uncertainty relation turns out to be an equality for variances of 3 independent observables in 2-dimensional Hilbert space, and pairwise inequalities are merely the corresponding projections of this equality, which looks enlightening for the understanding of the complementarity principle in QM.

## Methods

### General configurations of the Bloch vectors for variances

The generators of SU(*N*), represented as λ_*j*_, are *N*^2^ − 1 traceless Hermite matrices satisfying the following relation


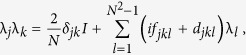


where *f*_*jkl*_ and *d*_*jkl*_ are the anti-symmetric and symmetric structure constants of SU(*N*). In term of Bloch vectors, the variance of a physical observable *A* takes the following form


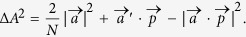


Here the new vector 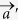 has the components of 
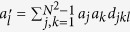
. We may define a new Hermitian operator 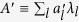. For given pair of observables *A* and *B*, there are the vector quaternary {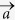, 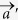, 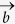, 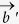}, where


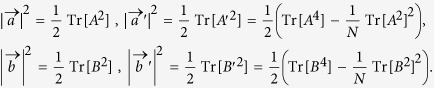


The angles among the set {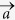, 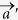, 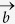, 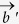} are all determined when *A* and *B* are given, i.e.,


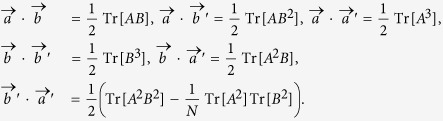


Similarly, when there are *k* observables in *N*-dimensional Hilbert space, 2*k* vectors in *N*^2^ − 1 real space are obtained with predetermined length and relative angles.

### An example of Proposition 1

Suppose the three observables in 2-dimensional Hilbert space are *A* = *σ*_1_, *B* = *σ*_2_, and *C* = *σ*_3_. For quantum state with 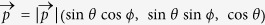, we have


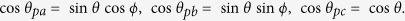


As cos^2^ *θ*_*pa*_ + cos^2^ *θ*_*pb*_ + cos^2^ *θ*_*pc*_ = 1, taking equations (7, 8, 9) we have


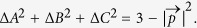


The sum of variances of *A*, *B*, *C* are 2 for pure states and 3 for completely mixed state.

### An example of *N*-dimensional system

For the sake of simplicity and illustration, here we present an example of state independent trade-off relations for two observables *A* and *B* of *N*-dimension with the Bloch vectors satisfying 
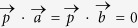
. This corresponds to the case of 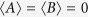. The variances now become


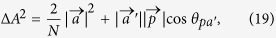



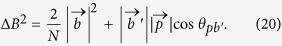


Along the same line as equation (11), we have the following trade-off relations between *A* and *B* for arbitrary state


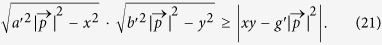


Here


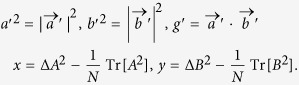


For completely mixed state where 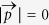, we have *x* = *y* = 0 from equation (21), and the variances reduce to Δ*A*^2^ = Tr[*A*^2^]/*N* and Δ*B*^2^ = Tr[*B*^2^]/*N*.

## Additional Information

**How to cite this article**: Li, J.-L. and Qiao, C.-F. Reformulating the quantum uncertainty relation. *Sci. Rep.* **5**, 12708; doi: 10.1038/srep12708 (2015).
